# Study protocol of a randomised controlled trial comparing perioperative intravenous insulin, GIK or GLP-1 treatment in diabetes–PILGRIM trial

**DOI:** 10.1186/1471-2253-14-91

**Published:** 2014-10-14

**Authors:** Jorinde AW Polderman, Peter L Houweling, Markus W Hollmann, J Hans DeVries, Benedikt Preckel, Jeroen Hermanides

**Affiliations:** Department of Anaesthesiology, Academic Medical Centre, Amsterdam, the Netherlands, Postbus 22660, 1100 DD Amsterdam, the Netherlands; Department of Anaesthesiology, Diakonessenhuis, Utrecht, the Netherlands, Bosboomstraat 1, 3582 KE Utrecht, the Netherlands; Department of Internal Medicine, Academic Medical Centre, Amsterdam, the Netherlands, Postbus 22660, 1100 DD Amsterdam, the Netherlands

**Keywords:** Diabetes mellitus type 2, Perioperative management, GLP-1 agonist

## Abstract

**Background:**

Diabetes mellitus (DM) is associated with poor outcome after surgery. The prevalence of DM in hospitalised patients is up to 40%, meaning that the anaesthesiologist will encounter a patient with DM in the operating room on a daily basis. Despite an abundance of published glucose lowering protocols and the known negative outcomes associated with perioperative hyperglycaemia in DM, there is no evidence regarding the optimal intraoperative glucose lowering treatment. In addition, protocol adherence is usually low and protocol targets are not simply met.

Recently, incretins have been introduced to lower blood glucose. The main hormone of the incretin system is glucagon-like peptide–1 (GLP-1). GLP-1 increases insulin and decreases glucagon secretion in a glucose-dependent manner, resulting in glucose lowering action with a low incidence of hypoglycaemia.

We set out to determine the optimal intraoperative treatment algorithm to lower glucose in patients with DM type 2 undergoing non-cardiac surgery, comparing intraoperative glucose-insulin-potassium infusion (GIK), insulin bolus regimen (BR) and GPL-1 (liragludite, LG) treatment.

**Methods/Design:**

This is a multicentre randomised open label trial in patients with DM type 2 undergoing non-cardiac surgery. Patients are randomly assigned to one of three study arms; intraoperative glucose-insulin-potassium infusion (GIK), intraoperative sliding-scale insulin boluses (BR) or GPL-1 pre-treatment with liraglutide (LG). Capillary glucose will be measured every hour. If necessary, in all study arms glucose will be adjusted with an intravenous bolus of insulin. Researchers, care givers and patients will not be blinded for the assigned treatment. The main outcome measure is the difference in median glucose between the three study arms at 1 hour postoperatively. We will include 315 patients, which gives us a 90% power to detect a 1 mmol l^−1^ difference in glucose between the study arms.

**Discussion:**

The PILGRIM trial started in January 2014 and will provide relevant information on the perioperative use of GLP-1 agonists and the optimal intraoperative treatment algorithm in patients with diabetes mellitus type 2.

**Trial registration:**

ClinicalTrials.gov, NCT02036372

## Background

It is expected that the worldwide prevalence of diabetes will increase from 220 million people now, to 300 million people in 2025 [1]. Type 2 diabetes mellitus (DM) is most common and accounts for 80% of the diabetic cases in the Western World [2]. Because DM is accompanied by macro- and microvascular complications, people with DM are more likely to be admitted to the hospital than people without DM. Twenty-two percent of in-hospital days are occupied by patients with DM, who are especially over-represented in the surgical population [3–5]. The prevalence of DM in hospitalised patients is up to 40% [3], thus the anaesthesiologist will encounter a patient with DM in the operating room on a daily basis.

Patients with DM have an increased risk of developing hyperglycaemia during and after surgery. The glycaemic control in the first 24 hours after surgery in patients with DM is poor and associated with an increased risk of postoperative infections [6–8]. In addition, hyperglycaemia is associated with increased risk of postoperative complications and length of hospital stay, irrespective of the diagnosis of DM [9–13].

Considering the high prevalence of DM in the operating theatre, the lack of evidence with regard to perioperative glucose regulation in non-cardiac surgery patients with DM is surprising. In contrast, stress hyperglycaemia in the ICU or in cardiac surgery patients has been investigated in a vast number of trials [14–16], but these studies did not focus on DM [17].

For the perioperative glucose regulation multiple protocols have been developed, ranging from intravenous glucose-insulin-potassium infusion to subcutaneous sliding-scale insulin bolus regimens. Despite this abundance of published glucose lowering protocols and the proven negative associations of perioperative hyperglycaemia in DM, there is no evidence to support an optimal perioperative glucose lowering treatment. In addition, DM protocol adherence is surprisingly low and glucose targets are frequently not achieved [18]. Considering only the postoperative period, the reduction of 1 mmol l^−1^ glucose in the RABBIT 2 surgery trial with a subcutaneous basal-bolus regimen compared to sliding scale algorithm significantly reduced postoperative complications in patients with DM type 2 [19]. These data also suggest that we cannot simply tolerate glucose levels above 10 mmol l^−1^.

Recently, incretins have been introduced to lower blood glucose. The main hormone of the incretin system is glucagon-like peptide–1 (GLP-1). GLP-1 increases insulin and decreases glucagon secretion in a glucose-dependent manner, resulting in low incidence of hypoglycaemia, which is a major advantage in the perioperative period and may reduce workload, thereby improving compliance. Intravenous administration of GLP-1 after major abdominal surgery normalised blood glucose levels, without causing hypoglycaemic events [20].

In conclusion, DM is a relevant and prevalent disease, which predisposes surgical patients to hyperglycaemia and postoperative complications. Despite being one of the most encountered co-morbidities in the operating theatre, there is a lack of clinical trials regarding the optimal perioperative management of elevated glucose levels. Our primary objective is to investigate the optimal intraoperative treatment algorithm to lower glucose in patients with DM type 2 undergoing non-cardiac surgery, comparing intraoperative GIK infusion, insulin bolus regimen and GPL-1 (liraglutide) treatment.

## Methods/Design

### Ethical approval

This study protocol was approved by the medical ethical committee of the Academic Medical Centre in Amsterdam and by the central committee on research involving human subjects (CCMO) acting as competent authority. The study protocol adheres to the Declaration of Helsinki and the guidelines of Good Clinical Practice (GCP). The study is registered at http://www.clinicaltrials.gov # NCT02036372.

### Trial design

**Figure 1:**
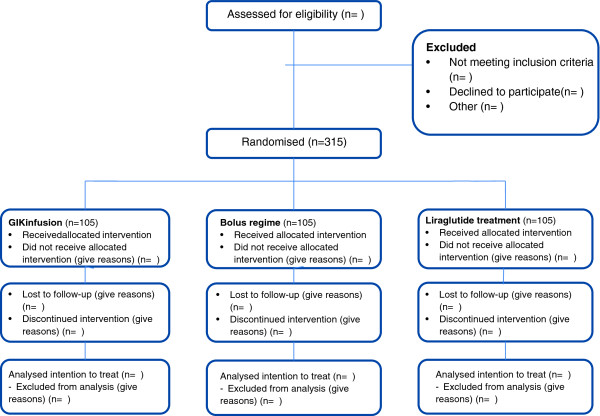
**Consort flow diagram of PILGRIM trial.**

The study is a multicentre open-label randomised controlled trial in adult patients with DM type 2, to evaluate the best treatment algorithm to lower glucose in the intraoperative setting, utilizing three parallel study arms. We will compare a glucose-insulin-potassium (GIK) infusion to an insulin bolus regimen and to treatment with the GLP-1 agonist liraglutide. The consort flow diagram of the trial is shown in Figure 1.

### Eligibility criteria

Patients with DM type 2, treated with oral glucose lowering tablets or a moderate dose of insulin, scheduled for non-cardiac surgery will be eligible for inclusion. This is also in concordance with the population studied in the RABBIT 2 surgery trial [19], showing a reduction in postoperative complications when lowering glucose by 1 mmol l^−1^. Further inclusion and exclusion criteria are listed below. We set a maximum of insulin dose per day, as we do not expect clinical effect from the GLP-1 agonist treatment when patients are treated with a daily dose of insulin >1 IU kg^−1^ bodyweight. Patients with a planned postoperative stay at the intensive care unit (ICU) will be excluded, as the participating ICU’s use a stricter glycaemic target, compared to the wards. Considering the suggested increased risk of pancreatitis when using GLP-1 analogues, patients with a history of acute or chronic pancreatitis are excluded. This point is more extensively addressed in the discussion section.

### Inclusion criteria

- Signed informed consent
- known diabetes mellitus type 2 for >3 months
- aged 18–75 years
- scheduled for elective non-cardiac surgery
- daily insulin dosage of <1 IU kg^−1^ body weight

### Exclusion criteria

- Oral corticosteroid use
- Treatment with long acting GLP-1 agonist or DDP-4 inhibitor
- Planned for day-care (ambulatory) surgery
- Planned ICU stay post-operatively
- Planned bowel surgery
- History of chronic pancreatitis or idiopathic acute pancreatitis
- Impaired liver function, defined as alanine aminotransferase (ALAT) ≥2.5 times upper normal limit
- Impaired renal function defined as serum-creatinine ≥133 μmol l^−1^ for males and ≥115 μmol l^−1^ for females
- Females of child bearing potential who are pregnant, breast-feeding or intend to become pregnant or are not using adequate contraceptive methods (adequate contraceptive measures as required by local law or practice)
- Known or suspected allergy to trial product(s) or related products
- Any condition that the local investigator feels would interfere with trial participation or the evaluation of results

### Study outline

The patients will be recruited during a pre-assessment visit at the anaesthesiology department, where written and oral information will be provided to each patient. Written informed consent will be obtained either at the pre-assessment visit or prior to surgery. For each patient, age, weight, length, relevant medical history and current medication use will be recorded. During the trial, prophylactic dexamethasone treatment for postoperative nausea and vomiting (PONV) is not allowed, as it is unknown to what extent dexamethasone influences glucose in patients with DM. The anaesthetic details will be noted and the grade of nausea and vomiting will be evaluated 1 hour before surgery and 4 hours after surgery using the PONV intensity scale [21] and numeric rating scale (NRS). Thirty days postoperatively, all patients will be called for the assessment of postoperative complications (Tables 1, 2 and 3) and we will review the charts of the patients. Patients will be called at least at three different dates and times to minimize bias due to lost to follow up. All the data will be entered in a digital, good clinical practice (GCP) approved database. During the whole study period, all (serious) adverse events will be recorded and reviewed by the Principal Investigator, according to the GCP guidelines. If it is determined that a medication related serious adverse event presents an unreasonable risk to patients, we will terminate the study or part of the study presenting that risk. The trial will be monitored by an independent monitor.

**Table 1:** **Composite endpoint major complications**

Major complications	Definition
Death	30- day mortality of any cause
Re-admission	Unplanned admission within 30 days after discharge
ICU-admission	Unplanned ICU-admission within 30 days after discharge
Re-operation	Unplanned surgical intervention within 30 days after discharge
Deep wound infection	CDC definition [23]
Pneumonia	CDC definition [23]
Sepsis/bacteremia	CDC definition [23]
Myocardial infarction	ECG changes/elevated troponine T and/or CK-MB enzymes
Cerebrovascular event	Diagnosed by CT-scan
Deep venous thrombosis	Diagnosed by Doppler and treatment started
Lung embolus	Diagnosed by spiral CT-scan
Stent thrombosis	Requiring surgical intervention
Bleeding	Requiring intervention or transfusion of RBC’s
Respiratory failure	Requiring intubation/ventilator assistance >24 hours
Renal failure	Requiring dialysis

ICU = intensive care unit, CDC = centre for disease control, CXR = chest X-ray, ECG = electrocardiogram, RBC = red blood cells.

**Table 2:** **Composite endpoint minor complications**

Minor Complications	Definition
Cystitis or urinary tract infection	CDC definition [23]
Superficial wound infection/wound leakage	CDC definition [23]/leakage of the wound requiring longer duration of hospital stay
Pancreatitis	Clinical diagnosis/Elevated amylase or lipase
Ileus	Lasting more than 72 hours
Delirium	Clinical diagnosis
Length of hospital stay	In days

CDC = centre for disease control.

**Table 3:** **Composite endpoint diabetes related complications**

Diabetes related complications	Definition
Hypoglycaemia	For which assistance was required
Diabetic Ketoacidosis	For which admission was required
Seeking medical help	Unplanned appointment with physician or DM nurse
Change in medication	Change in dose or medication stopped within 30 days of surgery
New medication	Additional diabetes medication started within 30 days of surgery

### Randomisation

If eligible, patients will be randomised into one of the three study arms. The randomisation will be 1:1:1 for each study arm, with stratification for insulin use. We will use block randomisation with random block sizes, ranging from 3 to 12. The randomisation will be performed with a computer based randomisation application (TENALEA Clinical Trial Data Management System). Patients and investigators are not blinded for the treatment allocation. All patients randomised will be included in the intention to treat analysis.

### Study procedures/interventions

In all patients, capillary glucose will be measured every 60 minutes, starting 30 min prior to surgery. The target range for plasma glucose for all study arms is 6–8 mmol l^−1^. An intravenous bolus of insulin will be administered according to treatment algorithm (Table 4). Capillary glucose will be measured using the Accu-Chek Inform (Roche diagnostics, Indianapolis, IL, USA).

**Table 4:** **Treatment algorithm**

Glucose measurement*	Insulin 1st bolus	If glucose increases after 1st bolus	If glucose increases after 2nd bolus
4-8 mmol/l	-	-	-
8-9 mmol/l	2 IU	4 IU	6 IU
9-10 mmol/l	3 IU	5 IU	7 IU
10-11 mmol/l	4 IU	8 IU	12 IU
11-12 mmol/l	5 IU	9 IU	13 IU
12-13 mmol/l	6 IU	12 IU	18 IU
13-14 mmol/l	7 IU	13 IU	19 IU
14-15 mmol/l	8 IU	15 IU	20 IU
15-16 mmol/l	9 IU	16 IU	21 IU
> 16 mmol/l**	10 IU	17 IU	22 IU

*If glucose is <4 mmol l^−1^, give 4 g glucose iv (20 ml glucose 20%) measure again after 10 minutes and consult research physician. If glucose is <2.3 mmol l^−1^ give 50 g glucose iv. (100 ml glucose 50%) measure again after 10 minutes and consult research physician. **Consult research physician. (multiply by 18 for mg dl^−1^).

Subjects in the glucose-insulin-potassium arm (GIK) will receive:

- The day before surgery, the evening dose of long acting insulin will be reduced with 50%.
- On the day of surgery, the regular insulin dose, if applicable, and the oral glucose lowering tablets will be withheld.
- GIK infusion: 10 mmol potassium-chloride and a calculated insulin dose are added to a 500 ml glucose 5% solution. The infusion is started 30 minutes before surgery at 83 ml/hr. The insulin dose in the GIK infusion will be calculated according to the formula:
  - ○ I = (PG-7)/(200/W) + 8 I = Insulin amount, PG = glucose 30 minutes preoperative, W = body weight in kg.
- Measure glucose every 60 minutes after start of surgery, start 30 min prior to surgery
  - ○ If glucose is 4–6 mmol l^−1^ stop infusion and measure again in 30 minutes
  - ○ If glucose is <4 mmol l^−1^, give 4 g glucose iv. (20 ml glucose 20%),
  - ○ measure again after 10 minutes and consult research physician.
  - ○ If glucose is <2.3 mmol l^−1^, give 50 g glucose iv. (100 ml glucose 50%),
    measure again after 10 minutes and consult research physician.
  - ○ If glucose is >8 mmol l^−1^, treat according to algorithm.

Subjects in the insulin Bolus Regimen arm (BR) will receive:

- The day before surgery, the evening dose of long acting insulin will be reduced by 50%.
- On the day of surgery:
  - ○ *If patients are using mealtime and longacting insulin/NPH*: mealtime morning dose will be withheld.
  - ○ *If patients are using only long acting insulin/NPH*: the dose of long-acting or NPH insulin will be reduced by 50%
  - ○ *If patients are using glucose lowering tablets*: glucose lowering tablets will be withheld on the morning of surgery.
- Measure glucose every 60 min, start 30 min prior to surgery
- Adjust glucose according to treatment algorithm

Subjects in the liraglutide arm (LG) will receive:

- On the day before surgery 0.6 mg liraglutide will be administered subcutaneously (s.c.) at 17.00 hr (5 pm). The dose of long acting and mealtime insulin will be reduced by 50% from the start of liraglutide treatment.
- On the day of surgery own insulin and oral glucose lowering tablets will be withheld.
- In case of nausea graded higher than minimal, the second gift of liraglutide will be omitted. Otherwise, treatment will be continued with 1.2 mg liraglutide s.c. on the morning of surgery.
- Measure glucose every 60 min, start 30 min prior to surgery
- Adjust glucose according to treatment algorithm

### Postoperatively

The assigned treatment will be continued up to 4 hours postoperatively. The patient will stay at the recovery room and will remain fasted. The treatment protocol will end 4 hours postoperatively. Hereafter, glucose will be monitored and treated according to the hospital protocol.

### Laboratory measurements

HbA1c, potassium and fasting serum glucose will be determined 1 h prior to surgery. Fasting serum glucose and potassium will be determined 1 hour, 4 hours and on day 1 postoperatively. When the patient has an arterial catheter during the surgical procedure, arterial glucose will be measured.

### Postoperative complications

The occurrence of postoperative complications will be assessed one month postoperatively. This will be determined via chart review and telephone contact. We will use a composite endpoint of postoperative complications derived from the RABBIT 2 surgery trial [19] and DelIT trial [22]. The definition of major and minor complications is shown in Tables 1 and 2. We used the definition of the centre for disease control (CDC) for the various infectious complications [23]. The occurrence of nausea and vomiting during hospital admission will be assessed and scored on the NRS scale. Diabetes related complications are shown in Table 3.

### Outcome measures

Our primary outcome measure is the difference in median serum glucose between the GIK, BR and LG arm 1 hour after surgery.

The secondary outcome measures are the difference in median serum glucose between the three study arms at 4 hours and 1 day postoperatively, the difference in the amount of insulin administered and difference in the occurrence of major, minor and diabetes related complications. the hourly measurements will be used to determine the perioperative area under the curve of the area outside the target range. In addition, the occurrence of mild or severe hypoglycaemia (glucose <4.0 mmol l^−1^ and <2.3 mmol l^−1^, respectively), hypokalaemia (<3.5 mmol l^−1^) or hyperkalaemia (>5.0 mmol l^−1^) is assessed. The difference in glucose variability, described as mean absolute glucose change (MAG), between study arms will be calculated [24]. The MAG is calculated by adding the absolute differences of the glucose values, divided by the time over which the measurements were taken.

### Statistical analyses

#### Sample size calculation

A 1 mmol l^−1^ decrease in glucose resulted in a relevant decrease in postoperative complications **(**8.7 mmol l^−1^ ± 1.8 and 9.7 ± 2.4) in the RABBIT 2 surgery trial [10]. We used the following formula to calculate the sample size: . Assuming a power of 90%, a significance level of 5% and a drop-out rate of 10%, we will need 105 patients per treatment group to detect a relevant difference of 1 mmol l^−1^ between treatment groups. Thus in total, we will need 315 patients.

### Analyses

Statistical analyses will be performed using SPSS version 21.0 (SPSS Inc., Chicago, IL, USA). All data will be analysed according to an intention-to-treat analysis. No interim analysis is planned. Glucose is not normally distributed, thus the between group difference in median glucose at 1 hour, 4 hours and 1 day postoperatively will be tested with the Kruskal-Wallis test and post-hoc testing using the Mann–Whitney *U* test. In case of missing glucose at one hour, the last glucose measured during surgery will be carried forward. Change in glucose will be analysed using repeated measurements ANOVA, with time as fixed effect and baseline glucose, time and the interaction between treatments as covariates. Furthermore a per-protocol analysis will be performed next to the intention-to-treat analysis. Finally, the between group differences of the secondary outcomes will be tested with the ANOVA, Kruskal-Wallis, student *t*, Mann–Whitney *U* and the Chi-square test, where appropriate.

## Discussion

Insulin infusion has been a long established regime for the treatment of DM in the perioperative period and is frequently combined with glucose and potassium for safety reasons [5, 25]. Moreover, a continuous infusion of glucose might reduce peripheral insulin resistance [26]. An alternative for the GIK infusion is an intravenous insulin bolus regime. Although no difference was seen in perioperative glucose control in insulin naïve- and insulin-dependent patients, an intravenous bolus regime might be less time consuming than a GIK infusion plus bolus regime [27, 28]. GLP-1 agonists have potential advantages over established DM treatments with insulin during the intraoperative period, due to the low risk of hypoglycaemia. For proper execution of all regimes, perioperative glucose measurements are mandatory.

Since the start of GLP-1 treatment, it has been suggested that the use of GLP-1 agonists is associated with an increased risk of pancreatitis [29]. However, patients with DM type 2 and obesity, regardless of treatment, have an increased risk of developing pancreatitis compared to patients without DM [30]. A recent meta-analysis of 55 randomized controlled trials (n = 33,350) showed no increased risk for pancreatitis in patients using GLP-1 agonists (OR 1.05, 95% CI 0.37 to 2.94) [31]. Also in a large observational trial with over 20,000 new incretin users, no increased risk for pancreatitis was found when compared to patients with DM using sulfonylureas, HR 1.00, 95% CI 0.59 to 1.70 [32]. Although these studies do not completely exclude the possibility of a slightly elevated risk of pancreatitis when using GLP-1 agonists, they are reassuring and an encouragement for more clinical trials using these new substances.

We think that the combination of perioperative use of GLP-1 agonists compared with more established perioperative treatment regimes, makes this a relevant trial on the optimal intraoperative treatment of DM during non-cardiac surgery.

## Abbreviations

DM: Diabetes mellitus
GIK: Glucose-insulin-potassium
BR: Bolus regime
LG: Liraglutide
GLP-1: Glucagon like peptide-1
ICU: Intensive care unit
CRF: Case report form
NPH: Neutral protamine Hagedorn
OR: Odds ratio
HR: Hazard ratio
CI: Confidence interval.
